# Phylogenetic Characterization of Rabies Virus Field Isolates Collected from Animals in European Russian Regions in 2009–2022

**DOI:** 10.3390/microorganisms11102526

**Published:** 2023-10-10

**Authors:** Sergei A. Chupin, Alexandr V. Sprygin, Nikolay G. Zinyakov, Nelly A. Guseva, Sergey V. Shcherbinin, Fedor I. Korennoy, Renat V. Adelshin, Ali Mazloum, Andrey Y. Sukharkov, Victoria V. Nevzorova

**Affiliations:** 1 Reference Laboratory for Rabies and BSE, Federal Centre for Animal Health, 600901 Vladimir, Russia; 2 Laboratory of Molecular and Genetic Researches, Federal Centre for Animal Health, 600901 Vladimir, Russia; spriginav@mail.ru (A.V.S.); ali.mazloum6@gmail.com (A.M.); 3 Reference Laboratory for Viral Avian Diseases, Federal Centre for Animal Health, 600901 Vladimir, Russia; 4 Information Analysis Centre under the Department for Veterinary Surveillance, Federal Centre for Animal Health, 600901 Vladimir, Russiakorennoy@arriah.ru (F.I.K.); 5 Irkutsk Anti-Plague Research Institute of Siberia and the Far East, 664047 Irkutsk, Russia; adelshin@gmail.com; 6 Faculty of Biology and Soil Sciences, Irkutsk State University, 664033 Irkutsk, Russia; 7 Belgorod Branch of the Federal Centre for Animal Health, 308015 Belgorod, Russia

**Keywords:** rabies, rabies virus, lyssavirus, phylogenetic analysis, European Russia

## Abstract

Rabies is a fatal disease of mammals that poses a high zoonotic risk to humans as well. The distribution of rabies is mainly driven by host animal migration and human-mediated dispersion. To contribute to the global understanding of the rabies virus (RABV) molecular epidemiology, 94 RABV field isolates collected from animals in 13 European Russian regions were phylogenetically characterized using the nearly full-size N gene nucleotide sequences. According to phylogenetic inferences, all isolates belonged to one of the two established phylogenetic groups, either group C (*n* = 54) or group D (*n* = 40), which are part of the clade *Cosmopolitan* of RABVs. Some representatives of group C collected from regions located far apart from each other had a remarkably high level of nucleotide identity. The possibility of the contribution of local bat species to the distribution of RABVs was discussed. Interestingly, over the years, the fraction of group D isolates has been constantly decreasing compared with that of group C isolates. The phylogenetic insights generated herein might have an important contribution to the control and surveillance of animal rabies epidemiology in the region.

## 1. Introduction

Rabies is a fatal and progressive zoonotic neurological infection caused by the rabies virus (RABV), which belongs to the species *Lyssavirus rabies*, genus *Lyssavirus*, subfamily *Alpharhabdoviridae*, family *Rhabdoviridae*, order *Mononegavirales*, class *Monjiviricetes*, subphylum *Haploviricotina*, phylum *Negarnaviricota*, kingdom *Orthornavirae*, and realm *Riboviria* [1,2]. It affects all warm-blooded animals, and the disease is prevalent worldwide except on some islands in areas such as Australia and Antarctica. Over 60,000 people die annually due to rabies, and approximately 15 million people receive postexposure rabies prophylaxis annually [1].

Over the last 10 years, according to the Ministry of Agriculture of the Russian Federation, the annual average number of animal rabies cases in Russia is 2100. However, the number of cases is decreasing. That is, 2533 and 2996 rabies cases were registered in 2012 and 2013, and the numbers of cases recorded in 2020 and 2021 were 1449 and 1032, respectively. According to the Federal Service for Surveillance, the number of human deaths in the same period was 2–7 cases per year. Moreover, 378,000 people received postexposure prophylaxis.

Wild animals are a basic reservoir and vector of the RABV [3]. If outbreaks occur, rabies infection spreads to domestic animals in the same area. To prevent further transmission of the RABV, domestic animals are vaccinated, and oral immunization is applied among wildlife. These strategies are considered effective [3]. However, the vaccine coverage and the scale of disease prevention measures are not sufficient to achieve a significant improvement in rabies status in the country [4].

Previous studies on the G gene nucleotide sequences have shown that the RABV isolates are split into two major phylogenetic lineages, namely, the bat- and dog-related RABV clusters [5]. The bat-related RABV lineage may be restricted to bats of the New World. Meanwhile, the dog-related RABV group contains viruses affecting dogs and wildlife carnivores in Europe, Africa, and Asia [5,6,7]. Phylogenetically, the dog-related phylogenetic group falls into six clades (the clades *Cosmopolitan*, *Africa 2*, *Africa 3*, *Arctic-related*, *Asian,* and *Indian Subcontinent*), where the group designation reflects the geographical range distributions [8,9].

Previously, several studies assessed the genetic characterization of Russian RABV field isolates, including those from European Russia [6,10,11,12]. Results showed the presence of RABVs belonging to the clades *Cosmopolitan* and *Arctic-related* in European Russia. The clades are classified into smaller divisions referred to as genetic groups. The European Russian viruses have been classified into genetic groups A, C, D and E, which are part of the clade *Cosmopolitan* [10]. However, the use of the partial and full-size N gene nucleotide sequences in the abovementioned publications was inconsistent. This caused an unclear and unvalidated understanding of RABV evolution, which inhibits the detailed understanding of rabies epidemiology in the area. In relation to this, gaining actual and systematic data on the full-size N gene can provide epidemiologically relevant insights into the RABV evolutionary processes and can delineate the migration pathways of the virus over vast territories in Russia. Furthermore, the list of regions in this part of the country where RABV has been genetically characterized is, by far, incomplete.

Therefore, the current study aimed to perform a phylogenetic analysis of 94 RABV field isolates collected from the European Russian territory in 2009–2022 based on the full-size N gene nucleotide sequences.

## 2. Materials and Methods

### 2.1. Samples

**Figure 1:**
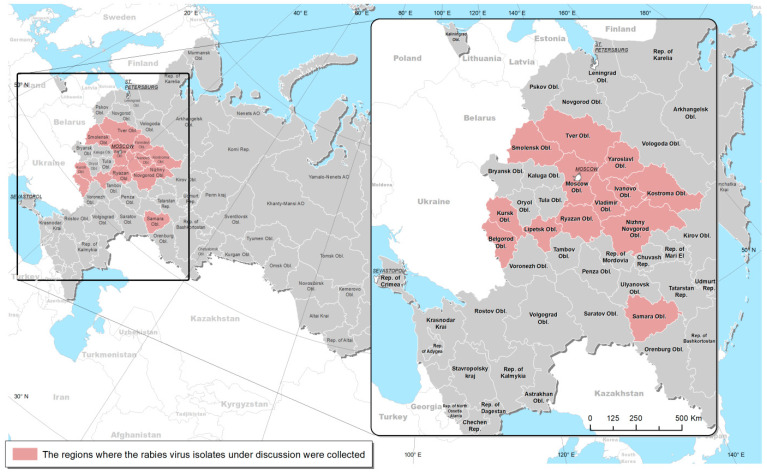
Russian regions where the rabies virus field isolates were collected.

To confirm rabies diagnosis, 94 brain tissue samples were collected from animals suspected of rabies in 13 European Russian regions from 2009 to 2022. The samples were then sent to the Reference Laboratory for Rabies and BSE of the Federal Centre for Animal Health (FGBI “ARRIAH”), Vladimir, Russia (Figure 1; Supplementary Table S1).

### 2.2. RNA Extraction and Reverse Transcription Polymerase Chain Reaction

Suspensions were prepared using the brain samples with phosphate-buffered saline. RNA extraction was performed using the RIBOsorb RNA extraction kit (FBIS Central Research Institute for Epidemiology, Moscow, Russia). RNA samples were used for immediate reverse transcription polymerase chain reaction (RT-PCR) or stored at −70 °C for further use. To detect the RABV genome, RT-PCR was performed using the method described in a previous study [13].

### 2.3. RABV N Gene Amplification and Sequencing

To sequence the full-size RABV N gene, two overlapping fragments corresponding to the RABV genome positions 20–787 and 653–1535 were synthesized for all isolates of interest using the following primers: NLeft1 (5′-RAGAAGAARCAGACAGCGTC-3′, forward primer) and NMidR3 (5′-CAGACCTGAACAGTCYTCATA-3′, reverse primer) for the first fragment, and NMidF1 (5′-GCTAATTGGAGTACTATACCG-3′, forward primer) and NRight1 (5′-GRTTGACGAARATCTTGCTC-3′, reverse primer) for the second fragment. Reverse transcription was primarily performed using the same primer pairs. RT-PCR was conducted with different reagents (Syntol, Moscow, Russia). Nucleotide sequencing was performed with the abovementioned primers and the BigDye Terminator v3.1 Cycle Sequencing Kit (Applied Biosystems) on the ABI Prism 3130 automatic sequencer.

Sequences were aligned with the CLUSTAL/W algorithm using the BioEdit software version 7.1.3.0, and the phylogenetic tree was built using the maximum likelihood algorithm based on the Tamura–Nei model and 1000 bootstraps with the MEGA 11 software [14]. With the help of the web service BLAST (Basic Local Alignment Search Tool, https://blast.ncbi.nlm.nih.gov/Blast.cgi (accessed on 30 March 2023)), a search was conducted for all nucleotide sequences of the full-size N gene nucleotide sequences of RABV that are significantly similar to the Russian isolates. Hence, closely related isolates collected from Poland and Kazakhstan were retrieved from GenBank and included in the tree.

The map of the Russian Federation at the first level of administrative division was drawn using the means of the ArcGIS Desktop 10.7 geoinformation system (ESRI, Redlands, CA, USA).

## 3. Results and Discussion

From 2009 to 2022, 94 field RABV isolates were collected from 13 regions in Russia (namely, Smolensk Oblast (Obl.), Kursk Obl., Belgorod Obl., Tver Obl., Moscow Obl., Lipetsk Obl., Yaroslavl Obl., Ivanovo Obl., Vladimir Obl., Ryazan Obl., Kostroma Obl., Nizhny Novgorod Obl., and Samara Obl.). Figure 1 shows the regions where the materials were collected. The presence of the RABV antigen in all samples was confirmed using the direct fluorescent antibody test. The full-size N gene nucleotide sequences of all isolates were determined via Sanger’s sequencing.

**Figure 2:**
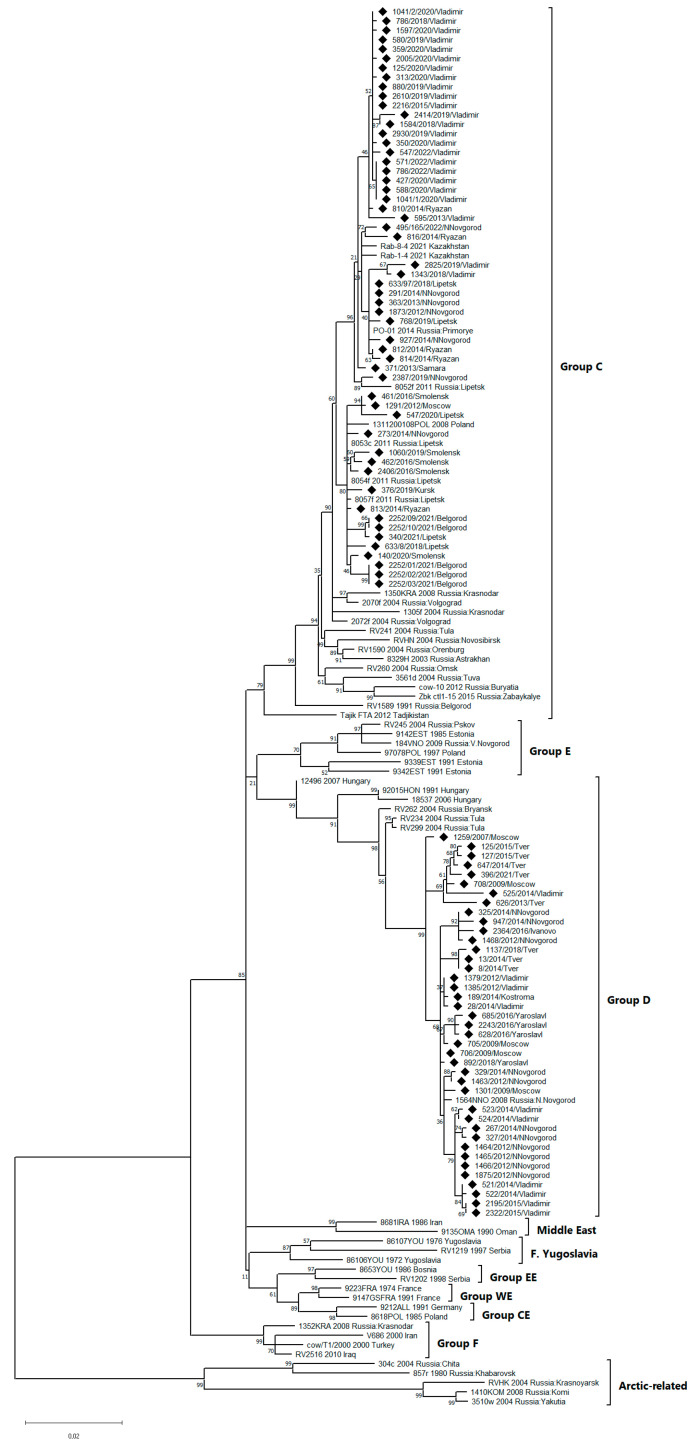
Phylogenetic tree built by analyzing the nucleotide sequences of the nearly full-size (1350 bp) N gene fragment (position in the N gene: 1–1350) of the RABV isolates. Legend: Square brackets indicate the genetic groups of the RABV. The diamonds mark the isolates in this study. The captions for the rest of the isolates indicate the name of the isolate and the year and country (region for Russian isolates) of detection. The bootstrap values for the key nodes are presented.

To build a phylogenetic tree, relevant isolates with full or nearly full-size (without the last three stop codon nucleotides) N gene sequences were used (Figure 2). All RABV isolates of interest, according to preliminary analysis, belonged to the genetic groups C and D. Thus, all previously described isolates detected in European Russia that belong to these two genetic groups were used. If there were two or more isolates collected in the same year in the same region that were genetically close to each other, only one of them was included in the tree.

To display the variety of RABVs circulating on the territory of Russia, some isolates belonging to other than the C or D genetic groups were included in the tree. Thus, isolates belonging to group A (also known as *Arctic*), group B (also known as *Arctic-like*), and group F (also known as *Caucasian* or *Iranian*) are also depicted on the tree.

In addition, for illustrative purposes, some representatives from the European (*Western European* [WE], *Central European* [CE], and *Eastern European* [EE]) and the Middle East genetic groups, as well as isolate 86107YOU collected from the former Yugoslavia, which is not a part of any described genetic groups, were also included in the tree.

Previous studies showed the presence of group C viruses in the following regions of European Russia: Belgorod Obl., Tula Obl., Krasnodar Krai, Volgograd Obl., Orenburg Obl. [10], Bryansk Obl., Voronezh Obl., Nizhny Novgorod Obl., Saratov Obl., Republic of Bashkortostan, Penza Obl. [11], Lipetsk Obl., and Republic of Dagestan [12].

Other studies have revealed the presence of group D viruses in the following regions of European Russia: Bryansk Obl., Tula Obl. [10], Tver Obl., Moskow Obl., Vladimir Obl., Ryazan Obl., and Nizhny Novgorod Obl. [11].

In this study, according to the phylogenetic tree topology, 54 RABV isolates belonged to the genetic group C and 40 isolates belonged to the genetic group D. Group C isolates were detected in the following regions: Smolensk Obl., Kursk Obl., Belgorod Obl., Moscow Obl., Lipetsk Obl., Vladimir Obl., Ryazan Obl., Nizhny Novgorod Obl., and Samara Obl.

The phylogenetic pattern of the group C field isolates on the tree corresponded to the locations of their origin. However, there are several uncommon exceptions. Some group C isolates collected far beyond the region of interest had an unexpectedly high level of relatedness to isolates obtained from European Russia.

The other analogous case was represented by two RABV isolates (Rab-8-4 and Rab-1-4) collected from cattle in Southern Kazakhstan in 2021. These isolates had a high level of identity (99.2–99.6%) with isolates in the central part of European Russia (Vladimir Obl., Ryazan Obl., Nizhny Novgorod Obl., and Lipetsk Obl.) and with the isolate PO-01 (99.6%) obtained from a brown bear in Primorsky Krai (far east of Russia) in 2014. Furthermore, two other isolates collected from Kazakhstan, although with 1023-bp sequences only, had a high level of identity (>99%) with the isolates obtained from the central part of European Russia and the Kazakh Rab-8-4 and Rab-1-4 isolates. The KZ(West)/cattle/5328(KT965737) isolate was collected from cattle in West Kazakhstan in 2014. Meanwhile, the KZ(West)/bat/111/2021 (OP585396) isolate was obtained from a bat (*Eptesicus serotinus*) in the same region in 2021 [15]. The close genetic relatedness of these two isolates and their localization in the same region indicate the possibility of virus transfer between terrestrial mammals and bats. This hypothesis can also explain the close relatedness of RABV isolates from Southern Kazakhstan and the central part of European Russia, which are remote from each other by >1500 km.

A considerable number of bats undergo significant seasonal movements between habitats [16]. During seasonal migrations, bats can overcome large distances within a relatively short time. Thus, a case of migration was documented after a bat (*Pipistrellus nathusii*) ringed in Latvia in 2015 was caught in Spain in 2017. That is, it traveled a distance of 2224 km [17]. In another case, a bat from the same species was ringed in the northeast of Russia (Vologda Obl.) and, in 63 days, was found in the French Alps (distance: 2486 km) [18]. Several species of the genus *Nyctalus* and the species *Vespertilio murinus* are among the other European long-distance migrants [16]. Bat migrations have been studied for a long time [19]. However, information about this phenomenon is still limited, and the migration routes have not been completely identified. In relation to this, large areas outside Europe and North America are still poorly explored [16].

The migration routes of bats in Europe have been evaluated relatively well [19]. In particular, the migration route of *Pipistrellus nathusii* from European Russia to Western Europe runs, among others, through the territory of Poland. Thus, in Poland, the discovery of the 1311200108POL isolate, which is significantly similar to the Russian isolates, may be a consequence of seasonal bat migration to a place of winter hibernation. The number of studies on the seasonal migration routes of bats from European Russia is insufficient. However, the wintering colonies of *P. nathusii* have been discovered in the Caucasus [20]. Considering that some bat species can overcome remarkable distances during seasonal migrations, the existence of bat colonies in European Russia, members of which migrate to places of winter hibernation in the territory of Kazakhstan, is quite possible. This hypothesis can explain the discovery of RABV isolates from Kazakhstan that have a high level of similarity with isolates from European Russia. This hypothesis is also supported by the fact that one of the Kazakh isolates was detected in a bat.

Bats are an important reservoir and vector of RABV in South and Central America [21]. On the contrary, in the Old World, bats are a reservoir and vector for most species of the genus *Lyssavirus* [22]. Meanwhile, the carriage of RABV by bats remained questionable until recently. The discovery of the KZ(West)/bat/111/2021 isolate in *Eptesicus serotinus* is probably among the first cases of such kind confirmed via genome sequencing [15].

The transfer of RABV in bats in the territory of Russia can well explain the two other cases of closely related viruses detected at considerable distances from each other.

Several RABV group C isolates from two regions in the central part of European Russia (Nizhny Novgorod Obl. and Lipetsk Obl.) were identical to the PO-01 isolate detected in a brown bear with full-size N gene sequences from Primorsky Krai (far east of Russia) in 2014. Furthermore, some RABV isolates from Vladimir Obl. and Ryazan Obl. differ only slightly from the PO-01 isolate (0.1–0.4%).

Currently, the genetic diversity of RABVs in Primorsky Krai has not been evaluated well. To the best of our knowledge, there is only a single study about one isolate (RV303) detected in a raccoon dog in 1979 (GenBank no. KY860613, [10]). This isolate belongs to the *Arctic-like* genetic group, the members of which considerably differ from those of group C. The authors who evaluated the PO-01 isolate [23] hypothesized that this isolate is a common representative of the RABV population in Primorsky Krai. However, there are data in contrast to this hypothesis. First, closely related and even identical isolates allegedly have circulated over the years in the territories that are situated 5000 km apart from each other, and this phenomenon is rare in RABV biology. Second, over several decades, only RABVs belonging to the genetic groups *Arctic*, *Arctic-like*, and group C subgroup, which are characteristic of Siberia, not of European Russia, have been detected in the far eastern region of Russia [24]. Some researchers [12] believe that the discovery of the PO-01 isolate in the far east was caused by an anthropogenic introduction, which seems to be likely. However, in light of the recently discovered data, there is no reason to completely deny the possibility of RABV transfer to the far east via bats.

The third case of surprisingly high relatedness is represented by the 1311200108POL isolate collected from a red fox in Poland that has a high level of identity (>99.0%) with Russian isolates. Despite the fact that many RABV isolates were detected and genetically characterized in different years in Poland [25], the 1311200108POL isolate has a significantly lower level of identity (<95.7%). This finding is also in accordance with the hypothesis of group C RABV transfer in bats. Further, this hypothesis is supported by the fact that group C viruses occupy unprecedentedly large areas (>5000 km), which is significantly larger than areas of the other genetic groups that are members of the clade *Cosmopolitan*. The members of this group are probably more adapted to bats compared with those of the other groups, and this allowed them to spread throughout wider areas. However, the hypothesis of anthropogenic transfer of group C rabies viruses cannot be completely rejected.

Forty newly characterized RABV isolates belonged to genetic group D. They originated from the following regions: Tver Obl., Moscow Obl., Yaroslavl Obl., Ivanovo Obl., Vladimir Obl., Kostroma Obl., and Nizhny Novgorod Obl.

All group D isolates characterized in this study form a fairly dense pool of isolates with a high level of relatedness (>98.0%). They somewhat differ from the isolates of the same group detected in Russia before 2008 (1.6–2.1%).

The distribution area of Russian group D viruses is significantly smaller than that of group C viruses. Group D viruses were registered in Tula Obl. and Bryansk Obl. of Russia and Hungary (with the isolates presented on the tree) and Ryazan Obl. of Russia (with the isolate not presented on the tree since only its short sequence is published [11]), in addition to the abovementioned regions. The 92015HON, 18537, and 12,496 isolates, which were detected in Hungary in 1991, 2006, and 2007, respectively, take a root position in this group, which infers their ancestral role. The isolates detected after 2004 in the central part of European Russia occupy an extreme position on the tree. Moreover, isolates detected in Bryansk Obl. and Tula Obl. (west of Russia) in 2004 take an intermediate position in the group.

This tree topology, in combination with the time and geography of origin of the group D isolates, clearly indicates that the distribution of group D viruses was observed in the direction from Eastern European countries, particularly Hungary, to the northeast and central regions of European Russia.

The detection rate of group D viruses is more likely to decrease. In some regions, group C viruses apparently substitute group D viruses. Thus, after 2015, in Vladimir Obl., none of the group D isolates have been detected. However, group C isolates have been identified regularly (22 isolates since 2015). Meanwhile, in 2015 and earlier years, 10 group D isolates and only 2 group C isolates were detected in the same region.

Similarly, in 2014 and earlier years, 17 isolates were detected in Nizhny Novgorod Obl., 12 of which belonged to group D and 5 to group C. After 2014, two isolates were detected in the region, and both of them belonged to group C. In Vladimir Obl. and Nizhny Novgorod Obl., this finding is the most graphic because of the relatively large number of isolates collected. In other regions, the tendency may be similar. Nevertheless, this notion is challenging to confirm because there is a smaller number of isolates collected from animals in these regions. Thus, further investigations should be performed to validate this finding.

## 4. Conclusions

Thus, with the help of phylogenetic inferences of the almost-full-size N gene sequences of 94 RABV field isolates detected in European Russia, these isolates were found to belong to the previously characterized genetic group C (*n* = 54) and group D (*n* = 40). In European Russia, the circulation area of group D viruses was significantly smaller than that of group C viruses. Group D viruses have spread apparently from the countries of Eastern Europe, in particular Hungary, to the northeast and central regions of European Russia. A partial substitution of group D viruses with group C ones was observed in some regions.

## Data Availability

The datasets generated during and/or analyzed during the current study are available from the corresponding author upon reasonable request. The gene sequences are available in GenBank.

## Supplementary Materials

The following supporting information can be downloaded at: https://www.mdpi.com/article/10.3390/microorganisms11102526/s1, Table S1: List of viruses used in the study [6,10,12,26,27,28,29,30,31,32,33].
