# Supplementary material for: Phylogenetic Characterization of Rabies Virus Field Isolates Collected from Animals in European Russian Regions in 2009–2022
Source: Microorganisms. 2023 Oct 10;11(10):2526. doi: 10.3390/microorganisms11102526 (PMC10609256; doi:10.3390/microorganisms11102526)
Supplement: Supplementary file 1 [file microorganisms-11-02526-s001.zip › microorganisms-2611227-supplementary.pdf]

microorganisms

MDPI

Supplementary Table S1. List of viruses used in the study

Country	Region/ Province (if available or relevant)	Species	Isolate	Year	Genetic group	Source/ reference	GB number
Russia	Nizhny Novgorod	red fox	495/165/2022/NNovgorod	2022	C	this study	OP311855
Russia	Vladimir	red fox	547/2022/Vladimir	2022	C	this study	OP311856
Russia	Vladimir	raccoon dog	571/2022/Vladimir	2022	C	this study	OP311868
Russia	Vladimir	red fox	786/2022/Vladimir	2022	C	this study	OP311858
Russia	Lipetsk	red fox	340/61/2021/Lipetsk	2021	C	this study	OP311852
Russia	Tver	raccoon dog	396/2021/Tver	2021	D	this study	OP311853
Russia	Belgorod	cat	2252/01/2021/Belgorod	2021	C	this study	OP311859
Russia	Belgorod	dog	2252/02/2021/Belgorod	2021	C	this study	OP311860
Russia	Belgorod	dog	2252/03/2021/Belgorod	2021	C	this study	OP311861
Russia	Belgorod	dog	2252/09/2021/Belgorod	2021	C	this study	OP311862
Russia	Belgorod	dog	2252/10/2021/Belgorod	2021	C	this study	OP311863
Russia	Vladimir	dog	125/2020/Vladimir	2020	C	this study	OP311864
Russia	Smolensk	cat	140/2020/Smolensk	2020	C	this study	OP311885
Russia	Vladimir	red fox	313/2020/Vladimir	2020	C	this study	OP311849
Russia	Vladimir	red fox	350/2020/Vladimir	2020	C	this study	OP311865
Russia	Vladimir	red fox	359/2020/Vladimir	2020	C	this study	OP311866
Russia	Vladimir	red fox	427/2020/Vladimir	2020	C	this study	OP311867
Russia	Lipetsk	red fox	547/2020/Lipezk	2020	C	this study	OP311883
Russia	Vladimir	red fox	588/2020/Vladimir	2020	C	this study	OP311884
Russia	Vladimir	red fox	1041/1/2020/Vladimir	2020	C	this study	OP311880
Russia	Vladimir	red fox	1041/2/2020/Vladimir	2020	C	this study	OP311881
Russia	Vladimir	cat	1597/2020/Vladimir	2020	C	this study	OP311882
Russia	Vladimir	red fox	2005/2020/Vladimir	2020	C	this study	OP311851
Russia	Kursk	red fox	376/2019/Kursk	2019	C	this study	OP311841

Russia	Vladimir	dog	580/2019/Vladimir	2019	C	this study	OP311889
Russia	Lipetsk	red fox	768/2019/Lipetsk	2019	C	this study	OP311891
Russia	Vladimir	red fox	880/2019/Vladimir	2019	C	this study	OP311842
Russia	Smolensk	dog	1060/2019/Smolensk	2019	C	this study	OP311843
Russia	Nizhny Novgo-rod	red fox	2387/2019/NNovgorod	2019	C	this study	OP311844
Russia	Vladimir	red fox	2414/2019/Vladimir	2019	C	this study	OP311888
Russia	Vladimir	red fox	2610/2019/Vladimir	2019	C	this study	OP311845
Russia	Vladimir	dog	2825/2019/Vladimir	2019	C	this study	OP311846
Russia	Vladimir	red fox	2930/2019/Vladimir	2019	C	this study	OP311847
Russia	Lipetsk	red fox	633/8/2018/Lipetsk	2018	C	this study	OP311833
Russia	Lipetsk	red fox	633/97/2018/Lipetsk	2018	C	this study	OP311834
Russia	Vladimir	elk	786/2018/Vladimir	2018	C	this study	OP311835
Russia	Yaroslavl	red fox	892/2018/Yaroslavl	2018	D	this study	OP311836
Russia	Tver	raccoon dog	1137/50/2018/Tver	2018	D	this study	OP311837
Russia	Vladimir	red fox	1343/2018/Vladimir	2018	C	this study	OP311839
Russia	Vladimir	goat	1584/2018/Vladimir	2018	C	this study	OP311896
Russia	Smolensk	dog	461/2016/Smolensk	2016	C	this study	OP328388
Russia	Smolensk	dog	462/2016/Smolensk	2016	C	this study	OP328389
Russia	Yaroslavl	red fox	628/2016/Yaroslavl	2016	D	this study	OP328391
Russia	Yaroslavl	red fox	685/2016/Yaroslavl	2016	D	this study	OP328392
Russia	Yaroslavl	red fox	2243/2016/Yaroslavl	2016	D	this study	OP328393
Russia	Ivanovo	cattle	2364/2016/Ivanovo	2016	D	this study	OP328399
Russia	Smolensk	dog	2406/2016/Smolensk	2016	C	this study	OP328390
Russia	Tver	raccoon dog	125/2015/Tver	2015	D	this study	OP328365
Russia	Tver	raccoon dog	127/2015/Tver	2015	D	this study	OP328366
Russia	Vladimir	red fox	2195/2015/Vladimir	2015	D	this study	OP328377
Russia	Vladimir	dog	2216/2015/Vladimir	2015	C	this study	OP328380
Russia	Vladimir	red fox	2322/2015/Vladimir	2015	D	this study	OP328378
Russia	Tver	dog	8/2014/Tver	2014	D	this study	OP328363

Russia	Tver	dog	13/2014/Tver	2014	D	this study	OP328364
Russia	Vladimir	red fox	28/2014/Vladimir	2014	D	this study	MF574196
Russia	Kostroma	red fox	189/2014/Kostroma	2014	D	this study	MF574197
Russia	Nizhny Novgorod	red fox	267/2014/NNovgorod	2014	D	this study	OP328368
Russia	Nizhny Novgorod	dog	273/2014/NNovgorod	2014	C	this study	OP328369
Russia	Nizhny Novgorod	red fox	291/2014/NNovgorod	2014	C	this study	OP328370
Russia	Nizhny Novgorod	red fox	325/2014/NNovgorod	2014	D	this study	MF574198
Russia	Nizhny Novgorod	red fox	327/2014/NNovgorod	2014	D	this study	MF574199
Russia	Nizhny Novgorod	red fox	329/2014/NNovgorod	2014	D	this study	MF574200
Russia	Vladimir	red fox	521/2014/Vladimir	2014	D	this study	OP328373
Russia	Vladimir	red fox	522/2014/Vladimir	2014	D	this study	OP328374
Russia	Vladimir	raccoon dog	523/2014/Vladimir	2014	D	this study	OP328376
Russia	Vladimir	dog	524/2014/Vladimir	2014	D	this study	OP328375
Russia	Vladimir	raccoon dog	525/2014/Vladimir	2014	D	this study	OP328379
Russia	Tver	dog	647/2014/Tver	2014	D	this study	OP328367
Russia	Ryazan	cat	810/2014/Ryazan	2014	C	this study	OP328383
Russia	Ryazan	cattle	812/2014/Ryazan	2014	C	this study	OP328384
Russia	Ryazan	cattle	813/2014/Ryazan	2014	C	this study	OP328385
Russia	Ryazan	cattle	814/2014/Ryazan	2014	C	this study	OP328386
Russia	Ryazan	dog	816/2014/Ryazan	2014	C	this study	OP328387
Russia	Nizhny Novgorod	dog	927/2014/NNovgorod	2014	C	this study	OP328371
Russia	Nizhny Novgorod	red fox	947/2014/NNovgorod	2014	D	this study	OP328372
Russia	Nizhny Novgorod	wolf	363/2013/NNovgorod	2013	C	this study	MF574191
Russia	Samara	red fox	371/2013/Samara	2013	C	this study	MF574184

Russia	Vladimir	cattle	595/2013/Vladimir	2013	C	this study	MF574194
Russia	Tver	raccoon dog	626/2013/Tver	2013	D	this study	MF574195
Russia	Moskow	red fox	1291/2012/Moscow	2012	C	this study	MF574185
Russia	Vladimir	red fox	1379/2012/Vladimir	2012	D	this study	MF574192
Russia	Vladimir	red fox	1385/2012/Vladimir	2012	D	this study	MF574193
Russia	Nizhny Novgorod	red fox	1463/2012/NNovgorod	2012	D	this study	MF574186
Russia	Nizhny Novgorod	dog	1464/2012/NNovgorod	2012	D	this study	MF574190
Russia	Nizhny Novgorod	cat	1465/2012/NNovgorod	2012	D	this study	MF574187
Russia	Nizhny Novgorod	red fox	1466/2012/NNovgorod	2012	D	this study	MF574188
Russia	Nizhny Novgorod	red fox	1468/2012/NNovgorod	2012	D	this study	MF574189
Russia	Nizhny Novgorod	cattle	1873/2012/NNovgorod	2012	C	this study	MF574182
Russia	Nizhny Novgorod	red fox	1875/2012/NNovgorod	2012	D	this study	MF574183
Russia	Moscow	red fox	705/2009/Moscow	2009	D	this study	OP328394
Russia	Moscow	red fox	706/2009/Moscow	2009	D	this study	OP328395
Russia	Moscow	red fox	708/2009/Moscow	2009	D	this study	OP328396
Russia	Moscow	raccoon dog	1301/2009/Moscow	2009	D	this study	OP328398
Russia	Moscow	dog	1259/2007/Moscow	2007	D	this study	OP328397
Russia	Veliky Novgorod	raccoon dog	184VNO	2009	E	[26]	JQ944704
Russia	Komi	reindeer	1410KOM	2008	A	[26]	JQ944707
Russia	Krasnodar	dog	1352KRA	2008	F	[26]	JQ944706
Russia	N.Novgorod	red fox	1564NNO	2008	C	[26]	JQ944708
Russia	Krasnodar	dog	1350KRA	2008	C	[26]	JQ944705
Russia	Krasnodar	red fox	1305f	2004	C	[10]	AY352461

Russia	Volgograd	red fox	2070f	2004	C	[10]	AY352484
Russia	Volgograd	red fox	2072f	2004	C	[10]	AY352485
Russia	Primorye	brown bear	PO-01	2014	C	[23]	KP997032
Russia	Chita	steppe fox	304c	2004	B	[10]	AY352459
Russia	Yakutia	wolf	3510w	2004	A	[10]	AY352486
Russia	Tuva	dog	3561d	2004	C	[10]	AY352481
Russia	Khabarovsk	raccoon dog	857r	1980	B	[10]	AY352458
Russia	Buryatia	cattle	cow-10	2012	C	[27]	JX423815
Russia	Zabaykalye	cattle	Zbk_ctl1-15	2015	C	[27]	KY243236
Russia	Lipetsk	red fox	Rus(Lipetsk)8052f	2011	C	[28]	KC595280
Russia	Lipetsk	cattle	Rus(Lipetsk)8053c	2011	C	[28]	KC595281
Russia	Lipetsk	red fox	Rus(Lipetsk)8057f	2011	C	[28]	KC595283
Russia	Lipetsk	red fox	Rus(Lipetsk)8054f	2011	C	[28]	KC595282
Russia	Belgorod	cat	RV1589	1991	C	[10]	AY352456
Russia	Orenburg	human	RV1590	2004	C	[10]	AY352472
Russia	Tula	dog	RV234	2004	D	[10]	AY352476
Russia	Tula	dog	RV241	2004	C	[10]	AY352477
Russia	Pskov	human	RV245	2004	E	[10]	AY352475
Russia	Omsk	red fox	RV260	2004	C	[10]	AY352465
Russia	Krasnoyarsk	human	RVHK	2004	A	[10]	AY352462
Russia	Novosibirsk	human	RVHN	2004	C	[10]	AY352463
Russia	Astrakhan	human	Rus(Astrakhan)_8329H_ 2003	2003	C	[12]	KT728348
Russia	Tula	red fox	RV299	2004	D	[10]	AY352479
Russia	Bryansk	red fox	RV262	2004	D	[10]	AY352457
Poland	Podlaskie voievodeship	red fox	1311200108POL	2008	C	unpublished	MF197741
Poland	-	raccoon dog	8618POL	1985	CE	[6]	U22840
Poland	-	raccoon dog	97078POL	1997	E	unpuplished	KX148154
Estonia	-	raccoon dog	9142EST	1985	E	[29]	U22476
Estonia	-	raccoon dog	9339EST	1991	E	[6]	U42707

Estonia	-	raccoon dog	9342EST	1991	E	[6]	U43432
Turkey	-	cattle	cow/T1/2000	2000	F	unpublished	DQ837474
Iraq	-	cattle	RV2516	2010	F	[30]	KF155000
Iran	-	cattle	V686	2000	F	[31]	AY854581
France	-	red fox	9223FRA	1974	WE	[6]	RVU43433
France	-	red fox	9147GSFRA	1991	WE	[6]	U22474
Germany	-	red fox	9212ALL	1991	CE	[6]	u22475
Iran	-	dog	8681IRA	1986	Mid-dle East	[29]	U22482
Sultanate of Oman	-	red fox	9135OMA	1990	Mid-dle East	[29]	U22480
Yugoslavia	-	red fox	86107YOU	1976	ND*	[6]	U42703
Yugoslavia	-	red fox	86106YOU	1972	ND	[29]	U22839
Serbia	-	red fox	RV1202	1998	EE	[32]	JF973787
Serbia	-	red fox	RV1219	1997	ND	[33]	MG458312
Bosnia	-	wolf	8653YOU	1986	EE	[6]	RVU42704
Tadjikistan	-	dog	Tajik FTA	2012	C	unpublished	KY765901
Hungary	-	human	92015HON	1991	D	unpublished	KX148160
Hungary	-	red fox	18537	2006	D	unpuplished	MK598338
Hungary	-	red fox	12496	2007	D	unpublished	MK598340
Kazakhstan	Turkistan	cattle	Rab-8-4	2021	C	unpublished	ON366708
Kazakhstan	Turkistan	cattle	Rab-1-4	2021	C	unpublished	ON366706

***ND - not defined**

References

26. Chupin S, Chernyshova E, Metlin A (2012) Complete genome sequence analysis of five Russian rabies virus field isolates. XXIII RITA - The Rabies in the Americas. P.78

27. Adelshin RV, Melnikova OV, Trushina YN et al (2015) A new outbreak of fox rabies at the Russian-Mongolian border. Virol Sin 30:313-5. https://doi.org/10.1007/s12250-015-3609-0
28. Poleshchuk EM, Deviatkin AA, Dedkov VG et al (2013) Complete genome sequences of four virulent rabies virus strains isolated from rabid animals in Russia. Genome Announc. 1(3):e00140-13. https://doi.org/10.1128/genomeA.00140-13
29. Kissi B, Tordo N, Bourhy H et al (1995) Genetic polymorphism in the rabies virus nucleoprotein gene. Virology 209:526-37. https://doi.org/10.1006/viro.1995.1285
30. Marston DA, McElhinney LM, Ellis RJ et al (2013) Next generation sequencing of viral RNA genomes. BMC Genomics 14:444. https://doi.org/10.1186/1471-2164-14-444
31. Nadin-Davis SA, Simani S, Armstrong J, Fayaz A, Wandeler AI (2003) Molecular and antigenic characterization of rabies viruses from Iran identifies variants with distinct epidemiological origins. Epidemiol Infect. 131:777-90
32. McElhinney LM, Marston DA, Freuling CM et al (2011) Molecular diversity and evolutionary history of rabies virus strains circulating in the Balkans. J Gen Virol. 92:2171-2180. https://doi.org/10.1099/vir.0.032748-0
33. Fischer S, Freuling CM, Müller T et al (2018) Defining objective clusters for rabies virus sequences using affinity propagation clustering. PLoSNegl Trop Dis 12:e0006182. https://doi.org/10.1371/journal.pntd.0006182

Disclaimer/Publisher's Note: The statements, opinions and data contained in all publications are solely those of the individual author(s) and contributor(s) and not of MDPI and/or the editor(s). MDPI and/or the editor(s) disclaim responsibility for any injury to people or property resulting from any ideas, methods, instructions or products referred to in the content.
